# Social factors and age play a significant role in cervical cancer and advanced-stage disease among Danish women

**DOI:** 10.1186/s12885-024-11994-4

**Published:** 2024-02-23

**Authors:** Sara Bønløkke, Jan Blaakær, Torben Steiniche, Maria Iachina

**Affiliations:** 1 Department of Clinical Medicine – Department of Pathology, Aarhus University, Aarhus N, Denmark; 2 Department of Pathology, Aarhus University Hospital, Palle Juul-Jensens Boulevard 99, Aarhus N, 8200 Denmark; 3 Department of Obstetrics and Gynaecology, Odense University Hospital, Odense C, Denmark; 4 Department of Clinical Research, University of Southern Denmark, Odense M, Denmark; 5 Department of Clinical Epidemiology, Odense University Hospital, Odense C, Denmark

**Keywords:** Epidemiology, Nationwide, Denmark, Social parameters, Cervical cancer screening, Non-attendance, Screening guidelines

## Abstract

**Background:**

For cervical cancer (CC), the implementation of preventive strategies has the potential to make cervical cancer occurrence and death largely avoidable. To better understand the factors possibly responsible for cervical cancer, we aimed to examine possible differences in age and social parameters as well as screening status between women with low- or high-stage cervical cancer and matched controls.

**Methods:**

Through the Danish Cancer Registry (DCR), women diagnosed with cervical cancer in Denmark between 1987 and 2016 were included. These were age- and residence-matched in a 1:5 ratio with controls from the general female population. The study population was sub grouped into a low-stage subpopulation with women with early-stage cervical cancer and matched controls and a high-stage subpopulation with women with late-stage cervical cancer and matched controls. Age and social parameters were compared within the subpopulations as well as between low- and high-stage cases. For part of the study population, screening attendance was examined to compare differences in adherence.

**Results:**

Overall, we found that the risk of cervical cancer is significantly increased in socially disadvantaged women and not least non-attenders in screening. Interestingly, the high-stage subpopulation was significantly older than the low-stage subpopulation (*p* < 0.001), and when examining the impact of age further, we found that for cervical cancer cases, the risk of having low-stage disease decreases significantly with increasing age, whereas the risk of having high-stage disease increases significantly with increasing age. In the screening cohort, significantly less cases than controls were attenders in screening with the most pronounced differences seen in the old subpopulation (women aged 50–64 years) and in the high-stage subpopulation (*p*-values all < 0.001). Interestingly, when examining the risk of CC for attenders and non-attenders, we demonstrated that many social parameters continue to influence the risk of cervical cancer, even in women attending screening.

**Conclusions:**

Older women, socially disadvantaged women, and non-attenders in screening are particularly vulnerable in terms of developing cervical cancer, especially high-stage disease. Therefore, improvements in the participating rate in screening as well as a revision of the current screening guidelines are needed.

**Supplementary Information:**

The online version contains supplementary material available at 10.1186/s12885-024-11994-4.

## Background

A persistent infection with carcinogenic human papillomavirus (HPV) genotypes is the cause of nearly all cases of cervical cancer (CC) [1]. This knowledge has opened new pathways for primary (prophylactic HPV vaccination) and secondary (cervical cancer screening and treatment of precancerous lesions) prevention [2]. HPV vaccination was introduced into the free-of-charge Danish childhood vaccination program in January 2009 for 12 year-old girls [3], and the HPV vaccination program currently has a 80% coverage among eligible girls (Girls born 2008) [4]. The implementation of HPV vaccination has caused a reduction in the incidence of HPV associated diseases in many countries [5–8]. Likewise, screening is estimated to reduce the CC incidence rate by 50–60% [9], and precancerous lesions can be treated, preventing progression to invasive disease [10–15]. In Denmark, cervical cancer screening was introduced in 1962 [16], and in 2007, it changed from being based on recommendations from the Danish Health Authorities to being an invitation of women aged 23–49 years every third year, and of women aged 50–64 years every fifth year [17]. After the introduction of cervical cancer screening, CC incidence decreased radically, but since year 2000, the incidence has remained stable [18] with approximately 350 Danish women being diagnosed with the disease each year [19], and with the incidence peaking in two age groups; women aged 35–44 years and women aged 75–84 years, respectively [20, 21].

Thus, the effectiveness of primary and secondary prevention is indisputable, and therefore, in order to reach the best protection against CC, high attendance in both HPV vaccination and cervical cancer screening is required. On the basis of this, World Health Organization (WHO) recently presented a roadmap for the period 2022–2030 to accelerate the elimination of CC as a public health problem in the WHO European Region [22]. It offers a vision for the path towards CC elimination in the Region by 2030 through universal access to HPV vaccination and appropriate cervical cancer screening and treatment services for the population. They describe a global strategy including three ambitious so-called 90-70-90 global targets aiming for the following: 90% of girls fully vaccinated with the HPV vaccine by the age of 15; 70% of women screened using a high-performance test by the age of 35, and again by the age of 45; and 90% of women with precancer treated, and 90% of women with invasive cancer managed.

Interestingly, previous data show that non-adherence to HPV vaccination is clearly associated with non-participation in cervical cancer screening [23–33], and furthermore, a large proportion of women with CC are non-attenders in cervical cancer screening, particularly women with more advanced disease stages [34–36]. Thus, to make an elimination of CC a reality, a better adherence to preventive strategies is essential. The aim of the present study was to examine possible differences in ageand social parameters as well as cervical cancer screening adherence between women with and women without a CC diagnosis, but also between women with early stages (localized disease according to ICD-10 [37]) and women with more advanced stages (regional disease or distant metastases according to ICD-10 [37]) of CC. A knowledge of these differences may contribute to a better understanding of the factors possibly responsible for the barriers to HPV vaccination and cervical cancer screening.

## Methods

### Setting and study population

**Fig. 1:**
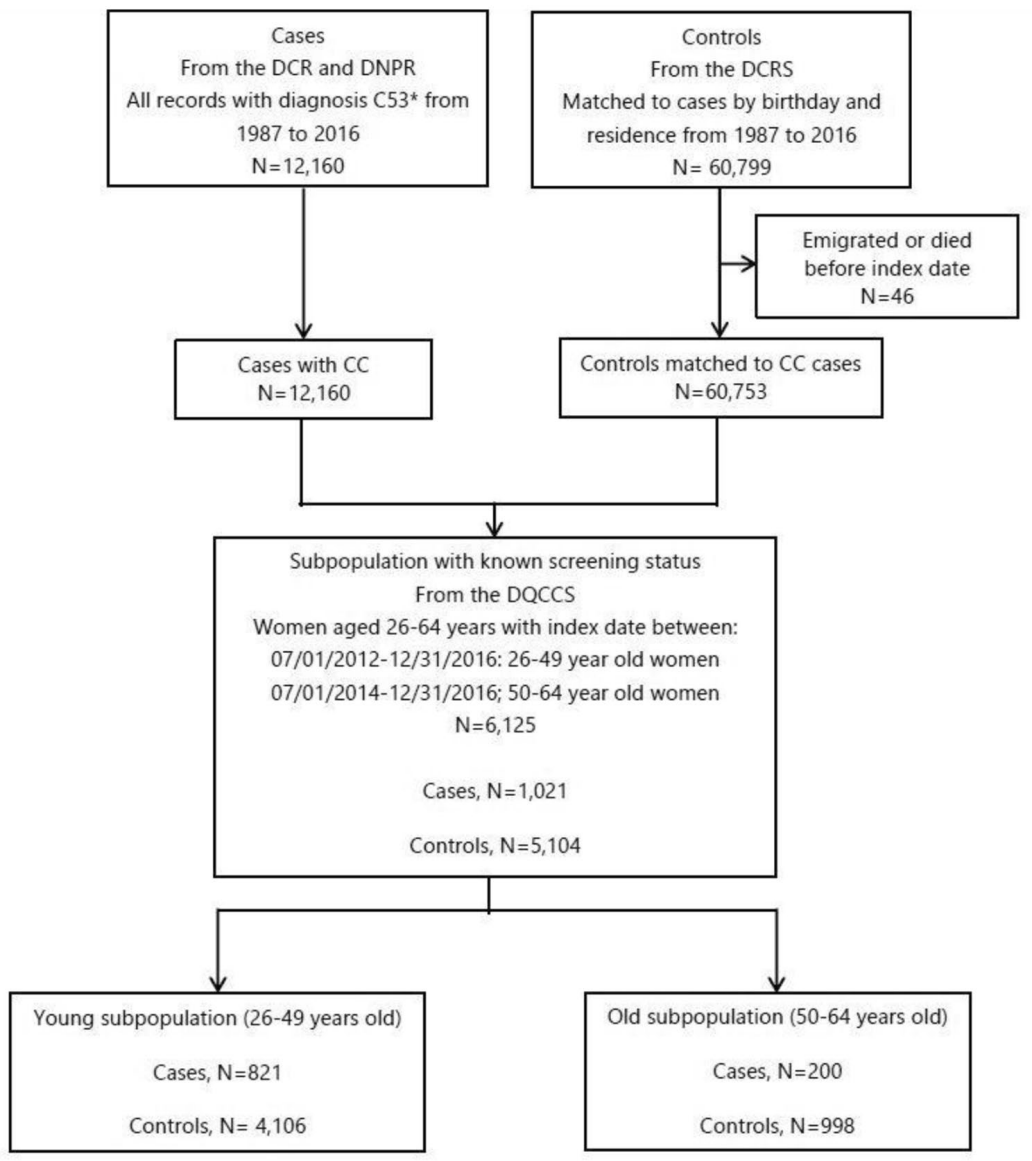
Selection of study population

This study is a population-based, nationwide, cohort study, where data were retrieved from different Danish National Health-care registries. Due to equal access to a centralized, tax-funded healthcare system for all residents in Denmark, a population-based study design was possible. Health service utilization is recorded in nationwide registries using each resident’s unique social security number (CPR number), which has been provided to all residents in Denmark since 1968 [38]. By means of the CPR number, cross-linkage of data on an individual level across registries is possible [39]. Thus, different registries were used to identify our study population; a case group with all CC patients diagnosed between 1987 and 2016, and a control group consisting of five matched women per case. First, we used the Danish Cancer Registry (DCR) to identify all women aged 18 years or more with a diagnosis of CC between 1987 and 2016. The DCR records information on all cancers diagnosed in Denmark, with registration being mandatory from 1987 [40]. Furthermore, DCR gives specific information on the stage of a cancer, were data from 1978 to 2003 has been converted from the modified ICD-7 classification to ICD-10 for diagnosis [37] making data from 1978 and onwards comparable. In most situations, the optimal control – to - case ratio is five [41], and therefore, for each CC patient, we identified five randomly matched women by year, date of birth (± 7 days), and area of residence at matching date (i.e., index date) through the Danish Civil Registration System (DCRS). To be in the control group, a person should [1] be alive and resident in Denmark at the time of the CC diagnosis for a matched patient from the case group (henceforth the ‘index date’); [2] not have a CC diagnosis in DCR; [3] and be matched only with one patient from the case group. The DCRS was furthermore used to access data on vital status and migration status for the study population. Figure 1 depicts the selection of the study population.

Information regarding relevant comorbidities up to ten years prior to the index date was retrieved from the Danish National Patient Registry (DNPR), and we classified these using the Charlson Comorbidity Index (CCI) (categorized as low [0], moderate [1 or 2], or high [> 2]) [42]. To ensure agreement of cancer diagnoses, incident CC cases were furthermore identified via DNPR, and these data were linked with the obtained data from the DCR. Since 1977, the DNPR contains information on all inpatient consultations according to the International Classification of Disease (ICD), and after 1995, the register contains information on all outpatient (ambulatory) hospital consultations as well [43]. Information on social parameters was retreated from Danish central registries. The *socio-economic status* of all women included was calculated using the information about personal equivalent disposable income in the year of the index date [44].The *socio-economic status* was defined as 0 - ‘low’ if the woman’s income was lower than the median of 213,993 DKK, and 1-high if the income was equal to or higher than the median. Education was calculated using information about the woman’s highest completed education and was defined in three categories: (i) 0 - low (municipal primary and lower secondary school), (ii) 1 - medium (upper secondary school), and (iii) 2 - high (higher education including bachelor, masters, and doctoral levels) [45]. Variable *civil status* was constructed using the household information, and it was defined as 0 - `yes’ if another adult was registered at the same address or 1- `no` if no other adult was registered at the same address [46]. Information about the woman’s working status in the year of the diagnosis was used to define the woman’s *social group;* (i) 0 - employed, (ii) 1 - senior citizen, or (iii) 2 - other.

Since 2007, the Danish national cervical cancer screening program has involved a screening invitation every third year for women aged 23–49 years and every fifth year for women aged 50–64 years [17]. The Danish Quality Database for Cervical Cancer Screening (DQCCS) monitors the quality of the nationwide screening program, and annual reports have been published since 2009 [47]. DQCCS was therefore used to collect information on screening attendance for women in our study population with an index date within a specific timeframe. Information on cervical smears in this database does not distinguish between smears collected for screening or smears collected as either a follow-up sample after previous detection of a precancerous lesion or as a part of the procedure for diagnosing CC, the last-mentioned in combination with a biopsy collection (i.e., colposcopy, biopsy, and cervical smear). Thus, since part of the diagnostic procedure includes collection of a cervical smear, cases from our total study population would most likely have a recent record of a smear prior to their diagnosis. Therefore, with the aim of only including and analyzing as many cervical smears collected for screening as possible, we decided to determine screening attendance as having a cervical smear taken more than 90 days prior to index date. Furthermore, since data in DQCCS is only available from year 2009 and onwards, and since screening is initiated at age 23, we included only part of the study population (i.e., subpopulation with known screening status) according to both index date and age at index date. Thus, to ensure that all women in this subpopulation would have been invited to screening prior to their index date and that sufficient screening history was available for all women, we only included women aged minimum 26 years and maximum 64 years. Furthermore, since some women attend screening with a reasonable delay after a screening invitation, we allowed for six months of delay, and thus, only women meeting the following criteria were included in the final subpopulation with known screening status: 26–49 year old women with an index date between 1st of July 2012 and 31st of December 2016, and 50–64 year old women with an index date between 1st of July 2014 and 31st of December 2016. Moreover, to ensure no inequality within the two age groups, only 3.5 years of screening history was included for all women in the young subpopulation, and only 5.5 years of screening history was included for all women in the old subpopulation, regardless of index date (Figure S1). By means of these criteria, 3.5 and 5.5 years of screening history prior to index date were available for the young and the old subpopulation, respectively. Controls comprised the same controls who were matched to the cases when they were included in the total study population. Furthermore, to enable determination of possible differences in screening attendance between women invited for screening every third year and women invited every fifth year, the subpopulation was subdivided into a young subpopulation aged 26–49 years at index date and an old subpopulation aged 50–64 years at index date (Fig. 1). Because the screening interval changes from every third to every fifth year at age 50, we made subanalyses excluding women aged 50–54 years from the analysis and thus ensuring that all women included in the two age cohorts have been invited for screening either within the last three or five years, respectively.

The Danish Vaccination Register (DDV) contains information on vaccinations reported by doctors and hospitals [48]. However, reporting of vaccinations was not mandatory until 15th of November 2015, and thus we were not able to gather information on HPV vaccination adherence in our study population. Furthermore, since HPV vaccination was not part of the free-of-charge Danish childhood vaccination program before 2009, only few from the study population would have been invited.

### Subgrouping of the study population

Treatment of CC depends on the stage of the disease [49], where women with localized disease are mainly treated with surgery and women with advanced disease with chemoradiation. Furthermore, the stage of the disease is the most important predictor of survival from CC, and the risk of death increases drastically with increasing disease stage [50]. Thus, to examine possible differences within the CC cases, information on the stage of the disease was retrieved from DCR [40], and the study population was sub-grouped into a low-stage subpopulation and a high-stage subpopulation, and separate analyses were performed on these subpopulations. Cases in the low-stage subpopulation included women with early-stage CC (localized disease according to ICD-10) and age- and residence-matched women from the overall control group. Cases in the high-stage subpopulation included women with advanced stage CC (regional disease or distant metastases according to ICD-10) and age- and residence-matched women from the overall control group.

Similarly, our subpopulation with known screening status was also sub-grouped into a low-stage subpopulation with known screening status and a high-stage subpopulation with known screening status.

### Statistical analyses

STATA version 17 (StataCorp, College Station, TX) was used for all statistical analyses. We used an approximation of the Fisher exact test for descriptive analyses. We performed logistic regression analyses adjusted for age, cohort and possible interaction between age and year of inclusion (cohort). Furthermore, to ensure that interaction between parameters are taken into account, strength of correlation between the explanatory parameters in a regression model were detected using a variance inflation factor (VIF). Here, a value of 1 indicates no correlation between a given explanatory variable and any other explanatory variable in the model, a value between 1 and 5 indicates moderate correlation but not severe enough to require attention, and a value greater than 5 indicates potentially severe correlation, making the coefficient estimates and *p*-values in the regression output unreliable. Furthermore, we conducted additional analyses by incorporating all relevant parameters as independent variables in a full model to investigate their impact on the outcome in both the total study population and the subpopulation with known screening status. We considered a *p*-value of ≤ 0.05 as statistically significant.

## Results

### Study population

In the total study population, we included 12,160 cases with CC and 60,753 matched controls (Fig. 1). After sub-grouping of cases according to disease stage, we identified 984 cases with unknown disease stage and the 4,904 controls matched to these cases, leaving 11.176 patients with known stage CC (case group) (median age at index date 50.19 years) and 55,849 age- and residence-matched references (median age at index date 50.16 years) (Table 1). The low-stage subpopulation included early-stage CC patients (localized disease, *n* = 6,126) (median age at index date 42.80 years) and age- and residence-matched controls (*n* = 30,618) (median age at index date 42.80 years), and the high-stage subpopulation included advanced stage CC patients (regional disease or distant metastases, *n* = 5,050) (median age at index date 61.23 years) and age- and residence-matched controls (*n* = 25,231) (median age at index date 61.21 years) (Table 1).

**Table 1:** Baseline characteristics for the study population

	Total study population^1^ (*n* = 72,913)		Sub-grouped study population with known disease stage (*n* = 67,025)			
			Low-stage subpopulation^2^ (*n* = 36,744)		High-stage subpopulation^3^ (*n* = 30,281)	
	Cases (*n* = 12,160)	Controls (*n* = 60,753)	Cases (*n* = 6,126)	Controls (*n* = 30,618)	Cases (*n* = 5,050)	Controls (25,231)
**Age**					**	**
< 35 35–44 45–54 55–64 65–74 > 75	1,996 (16.4%) 2,856 (23.5%) 2,148 (17.7%) 1,842 (15.2%) 1,758 (14.5%) 1,760 (12.8%)	9,964 (16.4%) 14,288 (23.5%) 10,745 (17.7%) 9,208 (15.2%) 8,774 (14.4%) 7,774 (12.8%)	1,489 (24.3%) 1,955 (31.9%) 1,136 (18.5%) 733 (12.0%) 542 (8.9%) 271 (4.4%)	7,433 (24.3%) 9,780 (31.9%) 5,685 (18.6%) 3,667 (12.0%) 2,700 (8.8%) 1,353 (4.4%)	356 (7.1%) 710 (14.1%) 872 (17.3%) 1,003 (19.9%) 1,056 (20.9%) 1,053 (20.9%)	1,776 (7.0%) 3,553 (14.1%) 4,362 (17.3%) 5,010 (19.9%) 5,277 (20.9%) 5,253 (20.8%)
**Cohort**					**	**
1987–1992 1993–1998 1999–2004 2005–2010 2011–2016	2,974 (24.5%) 2,578 (21.2%) 2,205 (18.1%) 2,206 (18.1%) 2,197 (18.1%)	14,858 (24.5%) 12,882 (21.2%) 11,012 (18.1%) 11,020 (18.1%) 10,981 (18.1%)	1,618 (26.4%) 1,253 (20.5%) 1,079 (17.6%) 1,048 (17.1%) 1,128 (18.4%)	8,088 (26.4%) 6,262 (20.5%) 5,389 (17.6%) 5,239 (17.1%) 5,640 (18.4%)	1,224 (24.2%) 1,159 (22.9%) 940 (18.6%) 937 (18.6%) 790 (15.6%)	6,116 (24.2%) 5,791 (22.9%) 4,694 (18.6%) 4,681 (18.6%) 3,949 (15.6%)
**Education**		*		*	**	**, *
Short Medium High Unknown	4,517 (37.2%) 4,010 (32.9%) 2,230 (18.3%) 1,403 (11.5%)	19,089 (31.4%) 20,911 (34.4%) 13,489 (22.2%) 7,264 (12.0%)	1,946 (31.8%) 2,350 (38.4%) 1,489 (24.3%) 341 (5.6%)	8,675 (28.3%) 11.779 (38.5%) 8,121 (26.5%) 2,043 (6.7%)	2,233 (44.2%) 1.340 (26.5%) 584 (11.6%) 893 7.7%)	8,881 (35.2%) 7.556 (29.9%) 4,365 (17.3%) 4,429 (17.6%)
**Comorbidity**					**	**
CCI = 0 CCI = 1 CCI = 2 CCI > 2	10,265(84.4%) 956 (7.9%) 650 (5.4%) 289 (2.4%)	51,793(85.3%) 4,601 (7.6%) 3,071 (5.1%) 1,288 (2.1%)	5,461 (89.1%) 355 (5.8%) 231 (3.8%) 79 (1.3%)	27,364 (89.4%) 1,718 (5.6%) 1,128 (3.7%) 408 (1.3%)	4,075 (80.7%) 507 (10.0%) 327 (6.5%) 141 (2.8%)	20,515 (81.3%) 2,390 (9.5%) 1,615 (6.4%) 711 (2.8%)
**Working status**		*		*	**	**, *
Working Senior Other Unknown	5,867 (48.3%) 4,608 (37.9%) 1,677 (13.8%) 8 (0.1%)	31,546 (51.9%) 21,361 (35.2%) 7,226 (11.9%) 620 (1.0%)	3,824 (62.4%) 1,327 (21.7%) 973 (15.9%) (0%)	19,493 (63.7%) 6,249 (20.4%) 4.533 (14.8%) 343 (1.1%)	1,648 (32.6%) 2,809 (55.6%) 592 (11.7%) (0%)	9,912 (39.3%) 12,858 (51.0%) 2,231 (8.8%) 230(1%)
**Socio-economic status**		*		*	**	**, *
Low High Unknown	6,555 (53.9%) 3,776 (31.1%) 1,829 (15.0%)	32,863 (54.1%) 23,338 (38.4%) 4,552 (7.5%)	3,171 (51.8%) 2,335 (38.1%) 620 (10.1%)	15,355 (50.2%) 12,665 (41.4%) 2,598 (8.5%)	2,865 (56.6%) 1,203 (23.8%) 982 (19.5%)	14,560 (57.7%) 9,070 (36.0%) 1,601 (6.4%)
**Civil status**		*		*	**	**, *
Living alone Living with a partner Unknown	4,613 (37.9%) 7,482 (61.5%) 65 (0.5%)	18,984 (31.3%) 40.993 (67.5%) 776 (1.3%)	1,919 (31.3%) 4,179 (68.2%) 28 (0.5%)	7,954 (26.0%) 22,210 (72.5%) 454 (1.5%)	2,256 (44.7%) 2,768 (54.8%) 30 (0.5%)	9,217 (36.5%) 15,751 (62.4%) 263 (1.0%)
**Born in Denmark**		*		*		**
Yes No	11,132 (91.6%) 1,028 (8.5%)	54,752 (90.1%) 6,001 (9.9%)	5,601 (91.4%) 525 (8.6%)	27,246 (89.0%) 3,372 (11.0%)	4,629 (91.7%) 421 (8.3%)	23,050 (91.4%) 2,181 (8.6%)

^1^ Total study population includes all women diagnosed with CC between 1987 and 2016, i.e., women with unknown stage of CC and matched controls are also included in this population

^2^ Low-stage subpopulation includes women diagnosed with early-stage CC (FIGO IA-IB2) between 1987 and 2016 and these women’s age- and residence-matched women from the overall control group

^3^ High-stage subpopulation includes women diagnosed with advanced stage CC (FIGO IIA+) between 1987 and 2016 and these women’s age- and residence-matched women from the overall control group

* Comparison between total cases vs. total controls, low-stage cases vs. low-stage controls, and high-stage cases vs. high-stage controls. χ²-test with 0.05 significance level

** Comparisons between low-stage cases vs. high-stage cases. χ²-test with 0.05 significance level

* Comparison between cases vs. controls *P* < 0.05

** Comparisons between low-stage cases vs. high-stage cases. *P* < 0.05

From the total study population (12,160 cases and 60,753 controls), we included 1,021 cases and 5,104 controls matched to these cases in the subpopulation with known screening status (*N* = 6,125), and these were sub-grouped according to age and afterwards according to disease stage (Fig. 1). Sub-grouping according to age resulted in 4,927 women aged 26–49 years in the young subpopulation and 1,198 women aged 50–64 years in the old subpopulation (Fig. 1). After sub-grouping of these two subpopulations according to disease stage, we identified 123 cases with unknown disease stage and the 615 controls matched to these cases, leaving 898 patients with known stage CC and 4,489 age- and residence-matched references. The low-stage subpopulation included early-stage CC patients (localized disease, *n* = 651) and matched controls (*n* = 3,255), and the high-stage subpopulation included advanced stage CC patients (regional disease or distant metastases, *n* = 247) and matched controls (*n* = 1,234).

### Risk of CC according to age and social parameters and screening attendance

Since cases with unknown stage CC may be a biased subpopulation, we analyzed baseline characteristics for both the total study population and the sub-grouped study population with known disease stage. For both populations, we showed some significant differences in the same social parameters between cases and controls (Table 1). Thus, for the total study population, significantly more cases had a short education, were living alone, and were born in Denmark (*p*-values all < 0.001), whereas significantly less cases were working, and had a high socio-economic status (*p*-values all < 0.001). When comparing women within the low- and the high-stage subpopulations, significant differences were observed in all the same parameters (*p*-values all < 0.001) with the most significant differences seen in the high-stage subpopulation. When comparing cases within the low- and high-stage subpopulations, our data show pronounced differences in almost all social parameters but also that high-stage cases were significantly older than low-stage cases (*p* < 0.001) (Table 1), highlighting a generally great difference in age between the low- and the high-stage subpopulation.

Even after adjusting for age, year of index date (cohort), and possible interaction between age and cohort, we found that the risk of getting CC remains influenced by various social parameters with significant differences according to level of education, working status, socio-economic status, civil status, and especially screening status (Table 2). In continuation of this, we showed that for CC cases, the same parameters influence the risk of having high-stage disease. These findings demonstrate that social parameters and screening status influence the risk of getting CC as well as the risk of having high-stage disease regardless of age. To ensure that interaction between parameters are taken into account, all parameters were further included in one model, and a VIF was used to detect strength of correlation between the explanatory parameters. Based on the whole study cohort, our results show that estimated VIF for all parameters were lower than 5, which means that it does not require extra attention. Moreover, using all possible combinations of parameters, our models showed that the change in estimated coefficients do not exceed one decimal regardless of the used combination of parameters. Overall, our findings show that the estimated effect of parameters were non-significantly different from the effect estimated for each parameter separately. This means that there is indeed interaction between parameters, but these parameters also independently influence on the risk of getting CC and of having high-stage disease. Nonetheless, acknowledging the potential correlation between social parameters, we conducted an additional analysis to assess the influence of each parameter while controlling for all other variables (i.e., education, comorbidity, working status, socio-economic status, civil status, country of birth, and age). Since all variables were only available for 59,288 women from the total study population (9,278 cases and 50,010 controls) and stage was only available for 8,635 women (5,200 in the low-stage subgroup and 3,435 in the high-stage subgroup), the analyses were only conducted on these women. Our findings show that the risk of getting CC is still influenced by education, socio-economic status, civil status, and country of birth. However, working status no longer has a significant impact on the risk (Table S1). In terms of the risk of having high-stage disease, our findings show that education, civil status, and age play a significant role, whereas working status and socio-economic status no longer have an impact (Table S1). In summary, the unadjusted and adjusted findings both demonstrate that various social parameters independently contribute to the risk of getting CC and of having high-stage disease.

As described earlier, screening attendance in the subpopulation with known screening status was defined as a screening sample taken more than 90 days prior to index date. For this subpopulation, we found that the risk of CC and especially high-stage disease is significantly increased in non-attenders compared to attenders in cervical cancer screening (OR for CC 2.85, 95% CI 2.37; 3.13 and OR for high-stage disease 2.76, 95% CI2.02; 3.76) (Table 2). Like with the total study population, a model including all parameters concluded that interaction between parameters is independent of the risk of CC.

**Table 2:** Risk of cervical cancer according to social parameters and screening attendance

	Risk of getting CC (total study population) (*n* = 72,913)	Risk of having high-stage disease (total cases with known stage) (*n* = 11,176)
	OR (95%CI)	OR (95%CI)
**Education**		
Medium vs. Short High vs. Short	0.76 (0.73; 0.81) 0.64 (0.61; 0.68)	0.71 (0.64; 0.78) 0.54 (0.47; 0.61)
**Comorbidity**		
CCI = 1 vs. CCI = 0 CCI = 2 vs. CCI = 0 CCI > 2 vs. CCI = 0	1.06 (0.98; 1.14) 1.07 (0.98; 1.17) 1.14 (1.00; 1.30)	1.13 (0.96; 1.32) 1.13 (0.93; 1.37) 1.12 (0.83; 1.52)
**Working status**		
Senior vs. Working Other vs. Working	1.34 (1.26; 1.43) 1.25 (1.17; 1.32)	1.61 (1.42; 1.82) 1.47 (1.30; 1.66)
**Socio-economic status**		
Low vs. High	1.27 (1.22; 1.33)	1.28 (1.17; 1.41)
**Civil status**		
Living alone vs. Living with a partner	1.37 (1.31; 1.43)	1.13 (1.03; 1.23)
**Born in Denmark**		
No vs. Yes	0.84 (0.78; 0.90)	1.08 (0.93; 1.25)
	Risk of getting CC (total subpopulation with known screening status) (*n* = 6,125)	Risk of having high-stage disease (total cases in subpopulation with known screening status and known stage) (*n* = 898)
**Screening attendance** ^**1**^		
No vs. Yes	2.85 (2.37; 3.13)	2.76 (2.02; 3.76)

Adjusted for age, cohort, and possible interaction

^1^ Screening attendance was only evaluated for part of the total study population, i.e., the subpopulation with known screening status, consisting of the following women from the population: 26–49 year old women with an index date between 1st of July 2012 and 31st of December 2016, and 50–64 year old women with an index date between 1st of July 2014 and 31st of December 2016

Nonetheless, it can be concluded from Table 2 that screening status has a substantial impact on a woman’s outcome. Consequently, it is highly probable that screening status itself is the parameter responsible for the detected effect on the social parameters analyzed in Tables 1 and 2 (i.e. education, comorbidity, working status, socio-economic status, civil status, and country of birth). Therefore, to determine the possible impact of screening status on our findings, we conducted additional analyses on the subpopulation with known screening status similar to our analyses on the total study population. Thus, like in Table 1 for the total study population, we examined baseline characteristics for the subpopulation with known screening status (Table S2). Similar to our findings in the total study population, there were significant disparities between cases and controls in education, working status, socio-economic status, civil status, and country of birth, and these differences were even more pronounced than in the total study population (Table S2). Additionally, after subgrouping the subpopulation according to screening status, similar differences were observed between cases and controls. Thus, for attenders, there were significant differences between cases and controls according to comorbidity index, working status, civil status and country of birth (*p*-values all < 0.001), whereas for non-attenders, education level, working status, civil status, and country of birth differed significantly between cases and controls (Table S2). Furthermore, for cases, attenders and non-attenders had significantly different education levels and working statuses (*p*-values all < 0.001), with a higher proportion of attenders having a high education level and being working. Conclusively, it seems that the only parameter probably slightly influenced by screening status is education.

**Fig. 2:**
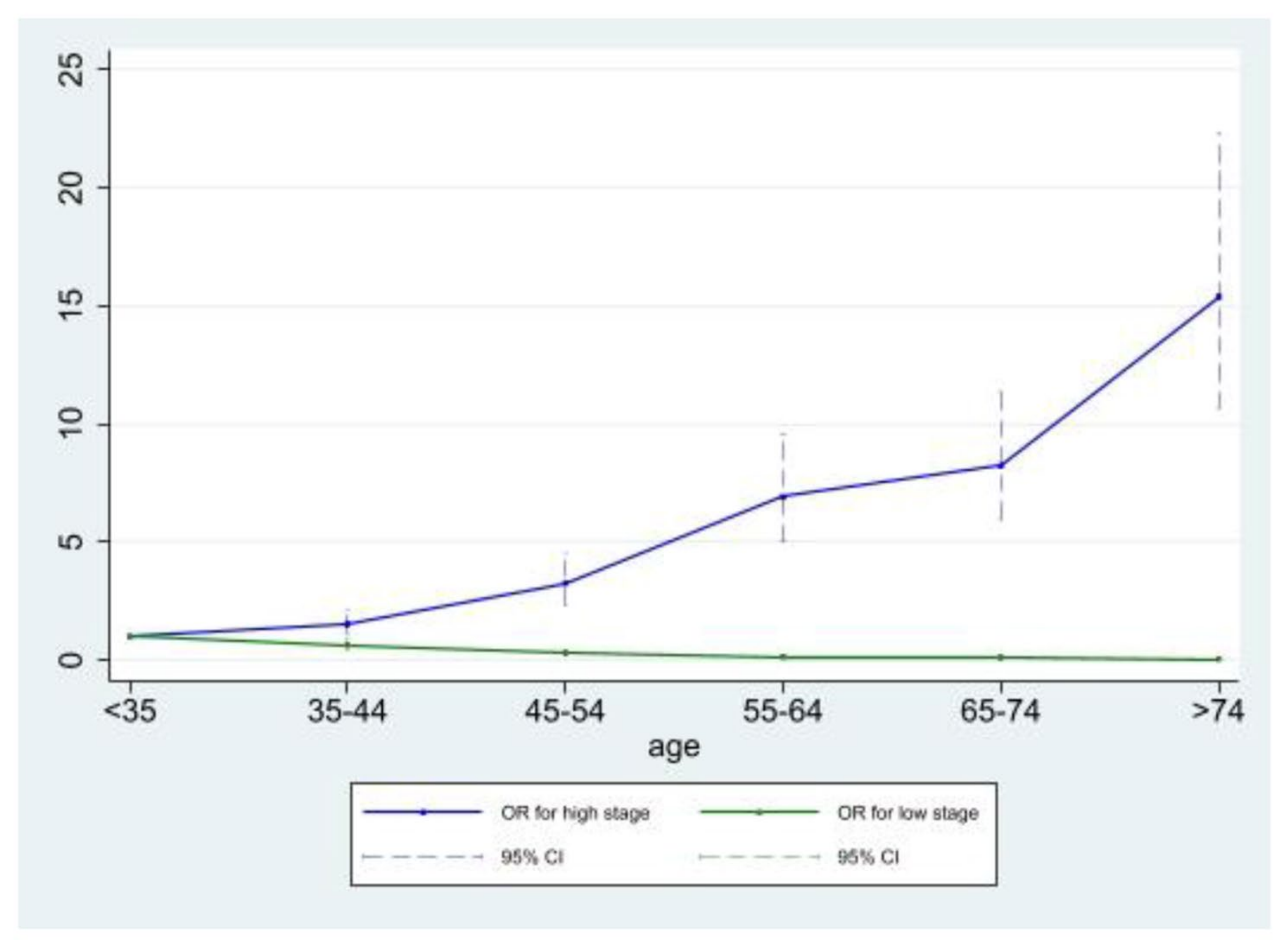
Risk of having low- or high-stage CC according to age The reference group consists of cases < 35 years

In Table 1, we saw a significant difference in age between the low- and the high-stage subpopulation. Thus, in CC cases, we studied the impact of age more closely by examining a possible difference in the risk of having low- or high-stage disease according to age (Fig. 2 and Table S3). When using women aged < 35 years as our reference group, we found that the risk of having low-stage disease decreases significantly with increasing age; from an OR of 0.65 (95% CI 0.47; 0.90) in the 35–44 year old age group to 0.06 (95% CI 0.04; 0.09) in the > 74 year old age group (Fig. 2 and Table S3). Furthermore, the risk of having high-stage disease increases significantly with increasing age; from an OR of 1.54 (95% CI1.11; 2.14) in the 35–44 year old age group to 15.40 (95% CI 10.64; 22.29) in the > 74 year old age group. In Denmark, CC incidence peaks in two age groups; in women around 35–44 years and in women around 75–84 years, respectively [20, 21]. Thus, our findings show that for women with CC, high-stage disease burden is predominantly carried by older women. We further found that the cohort (i.e. the year of index date) does not affect the risk of having low- or high-stage disease (Table S3), meaning that the risk of having low- or high-stage disease is not different in women diagnosed before compared to women diagnosed after the introduction of the nationwide recommendations for cervical cancer screening in 2007.

### CCS attendance according to age group and stage

For the total screening cohort, we found that significantly less cases than controls had previously attended screening, and this difference was even more pronounced in the high-stage subgroup (Table 3). Similarly, after having sub-grouped the screening cohort according to age, we found that for both for the young- and the old subpopulation, and the sub-grouped screening cohort according to disease stage, significantly less cases than controls had previously attended screening (*p*-values all < 0.001). The disparity was once again particularly evident in the high-stage subpopulation (Table 3), highlighting the low attendance of screening among women diagnosed with advanced stages of cervical cancer. Further, in both the young- and the old subpopulation, significantly more low-stage cases than high-stage cases had previously attended screening (*p*-values < 0.001) (Table 3). As previously described, we made a subanalysis excluding women aged 50–54 years, and results of this analysis show similar results on screening (data not shown).

**Table 3:** Screening attendance in the subpopulation with known screening status

	Total screening cohort^1^ (*n* = 6,125)		Sub-grouped screening cohort according to disease stage^2^ (*n* = 5,387)			
			Low-stage subpopulation (*n* = 3,906)		High-stage subpopulation (*n* = 1,481)	
**Screening attendance** ^**3**^ **26–64 years (yes/no)**						
	Cases (*n* = 1,021)	Controls (*n* = 5,104)	Low-stage cases (*n* = 651)	Low-stage controls (*n* = 2,715)	High-stage cases (*n* = 247)	High-stage controls (*n* = 1,234)
		*		*	**	*
Yes No	547 (53.6%) 474 (46.4%)	3,872 (75.9%) 1,232 (24.1%)	393 (60.4%) 258 (39.6%)	2,457 (75.5%) 789 (24.5%)	88 (35.6%) 159 (64.4%)	967 (78.4%) 267 (21.6%)
**Screening attendance according to age (yes/no)**						
	Cases (*n* = 821)	Controls (*n* = 4,106)	Low-stage cases (*n* = 553)	Low-stage controls (*n* = 2,765)	High-stage cases (*n* = 166)	High-stage controls (*n* = 831)
Young sub-population (26–49 years)		*		*	**	*
Yes No	446 (54.3%) 375 (45.7%)	3,129 (76.2%) 977 (23.8%)	331 (59.9%) 222 (40.1%)	2,095 (75.8%) 670 (24.2%)	60 (36.1%) 106 (63.9%)	654 (78.7%) 177 (21.3%)
	Cases (*n* = 200)	Controls (*n* = 998)	Low-stage cases (*n* = 98)	Low-stage controls (*n* = 490)	High-stage cases (*n* = 81)	High-stage controls (*n* = 403)
Old sub-population (50–64 years)		*		*	**	*
Yes No	101 (50.5%) 99 (49.5%)	743 (74.5%) 255 (25.6%)	62 (63.3%) 36 (36.7%)	362 (73.9%) 128 (26.1%)	28 (34.6%) 53 (65.4%)	313 (77.7%) 90 (22.3%)

^1^ Total screening cohort includes all cases and controls from the subpopulation with known screening status, including cases with unknown disease stage and these women’s controls

^2^ Sub-grouped screening cohort includes only cases from the total screening cohort with a known disease stage and these women’s controls

^3^ Screening attendance is defined as a screening sample taken more than 90 days prior to index date

* Comparison between total cases vs. total controls, low-stage cases vs. low-stage controls, and high-stage cases vs. high-stage controls. χ²-test with 0.05 significance level

** Comparisons between low-stage cases vs. high-stage cases. χ²-test with 0.05 significance level

* Comparison between cases vs. controls. *P* < 0.05

** Comparisons between low-stage cases vs. high-stage cases. *P* < 0.05

### Risk of CC in the subpopulation with known screening status

As described previously, screening status significantly influences the risk of getting CC (Table 2). Consequently, we further aimed to evaluate the risk of CC according to age and social parameters for non-attenders and attenders, respectively. Interestingly, for non-attenders, only civil status and country of birth significantly affected the risk of getting CC with an increased risk in women living alone and in women born outside Denmark (OR 1.28 (1.02; 1.16) and 2.64 (1.95; 3.59), respectively), whereas for attenders, working status, socio-economic status, civil status, and country of birth all significantly affected the risk of getting CC (Table S4). This shows that even though screening status significantly influence the risk of getting CC (Table 2), many of the same social parameters as described earlier (Tables 2 and 3) continue to influence the risk of CC, even in women attending screening. For CC cases, we showed that for both attenders and non-attenders, the risk of having high-stage disease was significantly increased in women ≥ 50 vs. women < 50 years (OR attenders 2.49 (1.47; 4.21) and non-attenders 3.08 (1.90; 4.99)) and in seniors vs. working women (OR attenders 2.29 (1.13; 4.63) and non-attenders 4.57 (2.35; 8.87)) but significantly decreased in women with high vs. women with short educations (OR attenders 0.49 (0.25; 0.97) and non-attenders 0.41 (0.23; 0.74) (Table S4). This corresponds well with our findings on CC cases in Fig. 2 showing that compared to women aged < 35 years, the risk of having high-stage disease increases significantly with increasing age. Like with the previous statistical analyses on both the total population and the subpopulation with known screening status (Table 2), all parameters were included in one model, and here, we found that interaction between parameters is independent of the risk of CC. Once again, we also conducted an additional analysis to assess the influence of each parameter while controlling for all other variables (i.e., education, comorbidity, working status, socio-economic status, civil status, country of birth, and age). All variables were only available for 6,222 women from the subpopulation (4,419 attenders and 1,706 non-attenders) and stage was only available for 898 women. The results from this additional analysis showed that the risk of CC is still influenced by working status, civil status, and country of birth, whereas socio-economic status of attenders no longer has a statistically significant impact. The risk of high-stage disease in CC cases is still significantly affected by age and working status, whereas education no longer reaches statistical significance.

### Differences in age and social parameters between attender- and non-attender controls

Since Table 2 showed a very significant impact of screening status in both the risk of CC and the risk of high-stage disease, we found it interesting to further examine, whether possible differences in baseline characteristics exist between attenders and non-attenders in cervical cancer screening. However, in our subpopulation with known screening status, a great proportion of CC cases would most likely belong to the non-attender group, and an inclusion of cases in these analyses would therefore introduce possible bias. Thus, with the aim of preventing this, potential differences in baseline characteristics between attenders and non-attenders were only examined for the controls from our subpopulation with known screening status (*N* = 5,104). Furthermore, these controls are Danish women matched to women with CC and may therefore not be completely representative for Danish women overall, and this must of course be taken into account. Nevertheless, we established that significantly more attenders than non-attenders had a medium or high education (44.1% vs. 39.1% and 41.3% vs. 31.1%, respectively), were working (79.3% vs. 60.8%), had a high socio-economic status (43.9% vs. 28.5%), were living with a partner (74.7% vs. 65.0%), and were born in Denmark (85.6% vs. 73.0%) (*p*-values all < 0.001) (Table S6). Similar results are found in the subanalysis excluding women aged 50–54 (data not shown). These parameters combined highly suggest the impact of different social parameters and not least country of birth on cervical cancer screening attendance.

## Discussion

To our knowledge, this is the first study to assess the association between age and social parameters and CC in a nationwide setting spanning almost three decades. Participation is a prerequisite for a screening program to succeed, and non-attendance has been shown to be the foremost risk factor for CC related to the screening program [14]. Nevertheless, the current screening coverage in Denmark is only 73% [51], and improvements in the participation rate in preventive strategies are therefore crucial. Thus, besides examining differences in age and social parameters between women with (cases) and women without (controls) CC as well as within the subpopulations (low-stage- and high-stage subpopulation, respectively), we furthermore examined the cervical cancer screening attendance in part of our study population (i.e., subpopulation with known screening status).

Overall, we found notable differences in social parameters between cases and controls, but also that the high-stage subpopulation was significantly older than the low-stage subpopulation. These findings emphasize that social status (i.e. education level, comorbidity level, working status, socio-economic status, civil status and country of birth) and age play an important role in regards to who develops CC and in what disease stage the cancer is detected. In continuation hereof, we found that compared to women aged < 35 years, the risk of having high-stage CC increases significantly with increasing age. These observations demonstrate that for the incidence of CC in Denmark, which has been shown to peak in women around 35–44 years and in women around 75–84 years, respectively [20, 21], the peak in older women is primarily driven by women diagnosed with high-stage disease. Previous studies have also reported this impact of social parameters and not least age. A Danish study found that low education, older age, and living alone are related to advanced CC stages due to non-attendance in cervical cancer screening [52]. Others suggest that CCs in less-advantaged women might be diagnosed at a more advanced stage because of low screening uptake, delay in seeking health care, and poor access to specialist care [53, 54]. After having adjusted for age, our findings support this by showing that the risk of getting CC and the risk of having advanced stage disease remains significantly increased in socially disadvantaged women and not least non-attenders in screening. The only rather unexpected finding is that the risk of CC is significantly decreased in non-natives compared to natives (OR 0.84, 95% CI 0.78; 0.90). One may speculate if this could be explained by different lifestyles or genetic differences between these two groups. However, this cannot be examined with the current data. In line with previous studies showing that most cases of CC develop in unscreened or under-screened women [14, 55, 56], we furthermore found a higher screening attendance among controls, and when comparing attenders and non-attenders in screening, our data show that significantly more non-attenders are less educated, not working, living alone, and more often non-natives compared to attenders. In line with these observations, findings from several studies from various countries and screening settings have underlined the influence of social parameters on the uptake of cervical cancer screening by showing an increased risk of being a non-attender in women with basic educational level or low income [57–63], in unmarried women [60–62, 64], and in women from ethnic minority backgrounds [65–67], specifically within the 50–64 year age group [68]. In continuation of this, others have shown that both HPV vaccination and cervical cancer screening coverages are considerably lower among non-natives and especially among women from non-western countries [33, 63, 69–72]. These evident findings on the impact of age, social status, and ethnicity on screening attendance underlines the importance of re-evaluating the current strategies for preventing CC, especially for these specific groups of women. This is supported by our finding that the risk of neither low-stage nor high-stage CC has changed significantly after the implementation of systematic nationwide screening in Denmark in 2007. However, it should be mentioned that some Danish counties had organized screening and relatively high coverage rates prior to 2007 [73], which may partly explain this, but nevertheless, it seems that screening behavior remains largely unchanged in the same type of women after the implementation of systematic nationwide screening. Prior studies have investigated the barriers to screening attendance specifically in older women, and a recent review suggests that these women cite embarrassment, an absence of symptoms, fear of pain, and bad experiences (including difficulties with the sample-taker accessing the cervix) as reasons for avoiding screening [74]. Another contributing factor is that many older women see themselves as being at lower risk of CC if they have had a single sexual partner for a long time or are no longer sexually active [75]. A recent qualitative study has examined how barriers to attending screening among older women (aged 50–64 years) from lower socio-economic and ethnic minority backgrounds may be broken down. They concluded that information designed specifically for older women should ensure that they understand the purpose and relevance of screening. Also, underlining changes to the programme that have made the experience less uncomfortable, and improved sample taker awareness of how women feel, may help to reduce the concerns related to previous negative experiences [76]. Furthermore, new screening technologies such as homebased HPV testing may increase screening participation by allowing women to use a self-sampling test kit at home. An overall positive effect on participation has been shown in several trials [77, 78], and a recent Danish study showed the benefits of homebased HPV testing especially in western immigrants and lower socioeconomic groups [79]. Thus, besides spreading the relevance of attending screening for these groups of women, we argue that an intensified effort including both more frequent and more specially designed screening invitations and follow-up reminders as well as an expansion of the use of self-sampling kits to a wider group of women may contribute to an improved screening participation rate among these vulnerable women.

In addition to improving the participating rate in women within the screening age, another very important factor to discuss is when to terminate cervical cancer screening. Current Danish cervical cancer screening guidelines advice women to stop screening at age 65 if they have an adequate prior normal result. However, recent findings question the appropriateness of these current guidelines [80, 81]. Reporting of CC incidence and mortality strongly relies on correction for hysterectomy [21, 82], and failure to do so may cause an underestimation of the true CC incidence [82–86], which may then mask the effect of preventive strategies. After controlling for hysterectomy, previous data show that CC incidence may not decline until at least age 85 [87] with the peak incidence seen in women aged 75–79 years [21]. Furthermore, older women attending screening are diagnosed at earlier stages than non-attending older women [88], and marked racial disparities exist in CC diagnoses over the age of 65 [89]. This vulnerability in older women in regards to developing CC is supported by results from the current study showing an increasing risk of high-stage disease with increasing age and further for attenders in cervical cancer screening, a significantly increased risk of getting high-stage disease in women aged 50–64 years compared to women aged < 50 years. These findings demonstrate that older women are more vulnerable in terms of developing high-stage CC, presumably due to difficulties with the sample-taker accessing the cervix of these post-menopausal women. Therefore, it seems that a revision of the current screening guidelines regarding when to terminate screening but not least improved screening techniques to ensure that viable material is collected from women of all ages is warranted.

## Conclusions

In spite of preventive strategies, the incidence of CC in Denmark has remained stable since year 2000. Here, we found that age, social status, and cervical cancer screening attendance significantly influence the risk of developing CC. Increasing age carries an increasing risk of CC and an increasing risk of having advanced stage disease. Additionally, socially disadvantaged women and non-attenders in cervical cancer screening are highly susceptible to the disease. These findings emphasize that in order to eliminate CC, different initiatives are needed, including; (i) specially designed initiatives to improve the participating rate in cervical cancer screening including e.g. by means of a more widespread use of homebased HPV testing, more frequent screening, and more specially designed screening invitations and, (ii) consider a revision of the current screening guidelines regarding when to terminate screening, and not least (iii) improved screening techniques to ensure that viable material is collected from women of all ages.

### Electronic supplementary material

Below is the link to the electronic supplementary material.

Supplementary Material 1

## Data Availability

The data that support the findings of this study are available from Statistics Denmark but restrictions apply to the availability of these data, which were used under license for the current study, and so are not publicly available. Data are however available from the authors upon reasonable request and with permission of the Danish Health Data Authority (contact person at the research service Anna Ruback Birkmose, anba@sundhedsdata.dk) and Statistics Denmark (contact person at the research service Marianne Andresen, mia@dst.dk).

## Abbreviations

HPV: Human papillomavirus
CC: Cervical cancer
WHO: World Health Organization
CPR number: Social security number
DCR: Danish Cancer Registry
DCRS: Danish Civil Registration System
DNPR: Danish National Patient Registry
CCI: Charlson Comorbidity Index
ICD: International Classification of Disease
DQCCS: Danish Quality Database for Cervical Cancer Screening
DDV: Danish Vaccination Register
VIF: Variance Inflation Factor
