# Supplementary material for: Social factors and age play a significant role in cervical cancer and advanced-stage disease among Danish women
Source: BMC Cancer. 2024 Feb 23;24:259. doi: 10.1186/s12885-024-11994-4 (PMC10893677; doi:10.1186/s12885-024-11994-4)
Supplement: Supplementary file 1 — Supplementary Material 1 [file 12885_2024_11994_MOESM1_ESM.docx]

**Supplementary material for “Social factors and age play a significant role in cervical cancer and advanced-stage disease among Danish women.”**

Sara Bønløkke^1, 2^, Jan Blaakær^3, 4^, Torben Steiniche^1, 2^, and Maria Iachina^5^

**Figure S1:** Determination of screening history for women in the subpopulation with known screening status

A: Young subpopulation


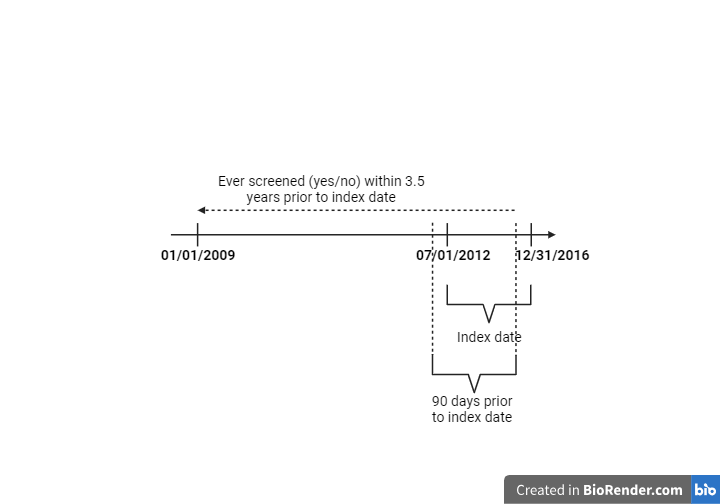


B: Old subpopulation


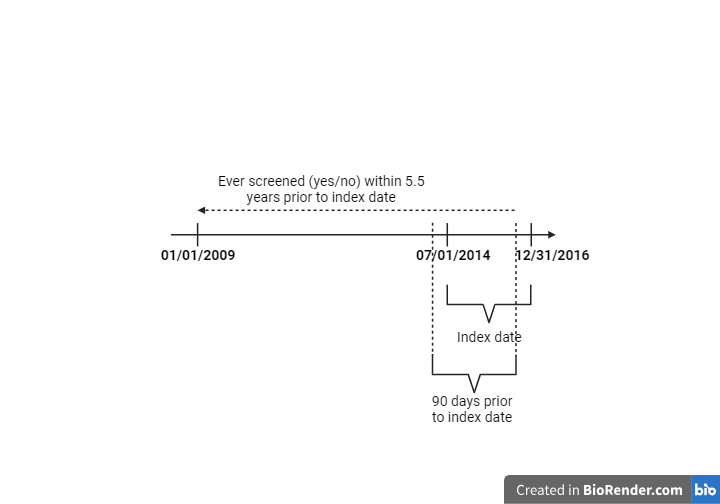


| Table S1 Risk of cervical cancer according to social parameters and screening attendance after controlling for all other variables^1^ | | |
| --- | --- | --- |
|  | Risk of getting CC  (total study population)  (n = 59,288) | Risk of having high-stage disease  (total cases with known stage)  (n = 8,635) |
|  | OR (95%CI) | OR (95%CI) |
| Education  Medium vs Short  High vs Short | 0.84 (0.79; 0.88)  0.73 (0.68; 0.78) | 0.85 (0.76; 0.95)  0.67 (0.58; 0.76) |
| Comorbidity  CCI = 1 vs CCI = 0  CCI = 2 vs CCI = 0  CCI >2 vs CCI = 0 | 1.03 (0.95; 1.12)  1.07 (0.96; 1.18)  1.04 (0.88; 1.23) | 1.13 (0.93; 1.36)  1.08 (0.87; 1.35)  0.88 (0.61; 1.27) |
| Working status  Senior vs Working  Other vs Working | 1.03 (0.95; 1.11)  1.07 (0.99; 1.14) | 1.13 (0.98; 1.32)  1.17 (1.02; 1.34) |
| Socio-economic status  Low vs High | 1.12 (1.07; 1.18) | 1.21 (0.99; 1.23) |
| Civil status  Living alone vs Living with a partner | 1.34 (1.27; 1.40) | 1.07 (0.97; 1.19) |
| Born in Denmark  No vs Yes | 0.76 (0.69; 0.83) | 1.17 (0.97; 1.40) |
| Age | 0.99 (0.98, 0.99) | 1.05 (1.04; 1.06) |

^1^ For each parameter, the parameter in focus was kept constant while controlling for all other variables (i.e., education, comorbidity, working status, socio-economic status, civil status, country of birth, and age)

| **Table S2.** Baseline characteristics for the subpopulation with known screening status  * Comparison between cases vs. controls. *P*<0.05  ** Comparisons between low-stage cases vs. high-stage cases or low-stage controls and high-stage controls. *P*<0.05. | | | | | | |
| --- | --- | --- | --- | --- | --- | --- |
|  | Total subpopulation^1^  (n = 6,125) | | Grouped subpopulation after screening attendance | | | |
|  |  |  | Attenders (n=4,419) | | Non-attenders (n=1,706) | |
|  | Cases  (n = 1,021) | Controls  (n = 5,104) | Cases (n=547) | Controls (n=3,872) | Cases  (n=474) | Controls (1,232) |
| **Age**  <35  35-44  45-54  55-64 | 237 (23.2%)  415 (40.7%)  240 (23.5%)  129 (12.6%) | 1,181 (23.1%)  2,082 (40.8%)  1,197 (23.5%)  644 (12.6%) | 130 (23.8%)  230 (42.1%)  122 (22.3%)  65 (11.9%) | 875 (22.6%)  1,609 (41.6%)  931 (24.0%)  457 (11.8%) | 107 (22.6%)  186 (39.0%)  118 (24.9%)  64 (13.5%) | **  306 (24.8%)  473 (38.4%)  266 (21.6%)  187 (15.2%) |
| **Education**  Short  Medium  High  Unknown | 199 (19.5%)  463 (43.4%)  342 (33.5%)  17 (1.7%) | *  769 (15.1%)  2,192 (43.0%)  1,982 (38.8%)  161 (3.1%) | 79 (14.4%)  249 (45.5%)  214 (39.1%)  5 (1.0%) | 488 (12.6%)  1.709 (44.1%)  1,599 (41.3%)  76 (2.0%) | **  120 (25.3%)  214 (45.2%)  128 (27.0%)  12(2.5%) | **, *  281 (22.8%)  483 (39.2%)  383 (31.1%)  85 (6.9%) |
| **Comorbidity**  CCI = 0  CCI = 1  CCI = 2  CCI >2 | 871 (85.3%)  78 (7.6%)  53 (5.1%)  19 (1.9%) | 4,499 (88.2%)  335 (6.6%)  203 (4.0%)  67 (1.3%) | 459 (83.9%)  48 (8.8%)  30 (5.5%)  10 (1.8%) | *  3,419 (88.3%)  254 (6.6%)  161 (4.2%)  38 (1.0%) | 412 (86.9%)  30 (6.3%)  23 (4.9%)  9 (2.0%) | **  1,080 (87.7%)  81 (6.6%)  42 (3.4%)  29 (2.4%) |
| **Working status**  Working  Senior  Other  Unknown | 720 (70.5%)  120 (11.8%)  181 (17.7%)  (0%) | *  3,820 (74.8%)  416 (8.2%)  756 (14.8%)  112 (2.2%) | 410 (75.0%)  54 (9.9%)  83 (15.2%)  (0%) | *  3,071 (79.3%)  255 (6.6%)  501 (12.9%)  45 (1.1%) | **  310 (65.4%)  66 (13.9%)  98 (20.7%)  (0%) | **, *  749 (60.8%)  161 (13.1%)  255 (20.7%)  67(5.4%) |
| **Socio-economic status**  Low  High  Unknown | 572 (56.0%)  336 (32.9%)  113 (11.1%) | *  2,532 (49.6%)  2,049 (40.1%)  523 (10.3%) | 294 (53.8%)  197 (36.0%)  56 (10.2%) | *  1,831 (47.3%)  1,698 (43.9%)  343 (8.9%) | 278 (58.7%)  139 (29.3%)  57 (12.0%) | **  701 (56.9%)  351 (28.5%)  180 (14.6%) |
| **Civil status**  Living alone  Living with a partner  Unknown | 348 (34.1%)  667 (65.3%)  6 (0.6%) | *  1,267 (24.8%)  3.695 (75.4%)  142 (2.8%) | 18X (33.6%)  36X (66.0%)  X (0.4%) | *  931 (24.0%)  2,894 (74.7%)  47 (1.2%) | 164 (34.6%)  306 (64.6%)  4 (0.8%) | **, *  336 (27.3%)  801 (65.0%)  95 (7.7%) |
| **Born in Denmark**  Yes  No | 913 (89.4%)  108 (10.6%) | *  4,214 (82.6%)  890 (17.4%) | 498 (91.0%)  49 (9.0%) | *  3,315 (85.6%)  557 (14.4%) | 415 (87.6%)  59 (12.5%) | **, *  899 (73.0%)  333 (27.0%) |

* χ²-test with 0.05 significance level.

X: according to the Danish Health Data authority, numbers less than three must not be shown.

| Table S3. Estimated Odds Ratio with 95% confidence Interval for having low- or high-stage disease for cervical cancer patients | | |
| --- | --- | --- |
|  | OR (95%CI) for low-stage disease | OR (95%CI) for high-stage disease |
| Age | | |
| 35-44 vs. <35 | 0.65 (0.47; 0.90) | 1.54 (1.11; 2.14) |
| 45-54 vs. <35 | 0.31 (0.22; 0.42) | 3.27 (2.36; 4.53) |
| 55-64 vs. <35 | 0.14 (0.10; 0.20) | 6.91 (5.00; 9.54) |
| 65-74 vs. <35 | 0.12 (0.09; 0.17) | 8.24 (5.96; 11.40) |
| >74 vs. <35 | 0.06 (0.04; 0.09) | 15.40 (10.64; 22.29) |
| Cohort (time of index date) | | |
| 1993 – 1998 vs. 1987 – 1992 | 0.71 (0.49; 1.02) | 1.41 (0.98; 2.02) |
| 1999 – 2004 vs. 1987 – 1992 | 0.63 (0.44; 0.92) | 1.58 (1.09; 2.29) |
| 2005 – 2010 vs. 1987 – 1992 | 0.80 (0.55; 1.17) | 1.24 (0.85; 1.81) |
| 2011 – 2016 vs. 1987 – 1992 | 0.85 (0.58; 1.26) | 1.17 (0.80; 1.72) |

| Table S4. Risk of cervical cancer according to social parameters in the subpopulation with known screening status | | | | |
| --- | --- | --- | --- | --- |
|  | Total screening cohort  (n=6,125) | | Screening cohort with known stage  (n=898) | |
|  | Risk of getting CC  (attenders)  (n=4,419) | Risk of getting CC (non-attenders)  (n=1,706) | Risk of having high-stage disease  (case attenders)  (n=481) | Risk of having high-stage disease  (case non-attenders)  (n=417) |
|  | OR (95%CI) | OR (95%CI) | OR (95%CI) | OR (95%CI) |
| Age  Age≥50 vs age<50 | 0.95 (0.75; 1.20) | 1.01 (0.78; 1.31) | 2.49 (1.47; 4.21) | 3.08 (1.90; 4.99) |
| Education^1^  Medium vs short  High vs short | 0.89 (0.67; 1.17)  0.81 (0.61; 1.07) | 1.03 (0.79; 1.35)  0.78 (0.58; 1.05) | 0.72 (0.38; 1.36)  0.49 (0.25; 0.97) | 0.62 (0.37; 1.03)  0.41 (0.23; 0.74) |
| Comorbidity^1^  CCI = 1 vs CCI = 0  CCI = 2 vs CCI = 0  CCI >2 vs CCI = 0 | 1.43 (1.04; 1.98)  1.43 (0.95; 2.15)  2.03 (1.00; 4.13) | 0.96 (0.62; 1.49)  1.41 (0.83; 2.40)  0.80 (0.37; 1.72) | 1.17 (0.55; 2.51)  1.56 (0.60; 4.02)  1.23 (0.22; 6.75) | 3.20 (1.37; 7.52)  0.48 (0.17; 1.35)  Can not be estimated |
| Working status^1^  Senior vs Working  Other vs Working | 1.70 (1.23; 2.37)  1.22 (0.94; 1.57) | 0.99 (0.71; 1.38)  0.93 (0.71; 1.21) | 2.29 (1.13; 4.63)  1.09 (0.55; 2.17) | 4.57 (2.35; 8.87)  2.14 (1.29; 3.54) |
| Socio-economic status^1^  Low vs high | 1.38 (1.14; 1.67) | 0.99 (0.78; 1.27) | 1.36 (0.81; 2.27) | 1.64 (1.02; 2.65) |
| Civil status^1^  Living alone vs living with a partner | 1.59 (1.31; 1.93) | 1.28 (1.02; 1.61) | 1.05 (0.65; 1.73) | 1.17 (0.76; 1.80) |
| Born in Denmark^1^  No vs Yes | 1.71 (1.26; 2.33) | 2.64 (1.95; 3.59) | 0.55 (0.27; 1.14) | 0.96 (0.52; 1.75) |

^1^ Adjusted for age and cohort

| Table S5. Risk of cervical cancer according to social parameters in the subpopulation with known screening after controlling for all other parameters^1^ | | | | |
| --- | --- | --- | --- | --- |
|  | Total screening cohort  (n= 5,438) | | Screening cohort with known stage  (n= 784) | |
|  | Risk of getting CC  (attenders)  (n=3,990) | Risk of getting CC (non-attenders)  (n=1,448) | Risk of having high-stage disease  (case attenders)  (n=429) | Risk of having high-stage disease  (case non-attenders)  (n=355) |
|  | OR (95%CI) | OR (95%CI) | OR (95%CI) | OR (95%CI) |
| Age  Age≥50 vs age<50 | 0.90 (0.80; 1.00) | 0.94(0.82; 1.07) | 1.38 (1.02; 1.86) | 1.63 (1.25; 2.12) |
| Education  Medium vs short  High vs short | 0.96 (0.71; 1.30)  0.94 (0.68; 1.29) | 1.04 (0.76; 1.41)  0.86 (0.60; 1.27) | 0.88 (0.42; 1.85)  0.63 (0.28; 1.44) | 0.88 (0.47; 1.65)  0.74 (0.36; 1.58) |
| Comorbidity  CCI = 1 vs CCI = 0  CCI = 2 vs CCI = 0  CCI >2 vs CCI = 0 | 1.43 (1.01; 2.02)  1.34 (0.87; 2.06)  1.83 (0.87; 3.85) | 0.87 (0.54; 1.38)  1.13 (0.62; 2.05)  0.96 (0.43; 2.12) | 1.18 (0.52; 2.68)  1.11 (0.37; 3.32)  0.96 (0.17; 5.58) | 2.08 (0.79; 5.51)  0.16 (0.03; 0.77)  Can not be estimated |
| Working status  Senior vs Working  Other vs Working | 1.48 (1.02; 2.18)  1.15 (0.85; 1.55) | 0.82 (0.55; 1.21)  0.92 (0.66; 1.27) | 1.74 (0.73; 4.10)  0.94 (0.42; 2.12) | 2.77 (1.21; 6.36)  1.79 (0.95; 3.36) |
| Socio-economic status^1^  Low vs high | 1.15 (0.93; 1.44) | 0.99 (0.74; 1.30) | 1.10 (0.59; 2.02) | 1.17 (0.67; 2.05) |
| Civil status  Living alone vs living with a partner | 1.54 (1.25; 1.92) | 1.20 (0.93; 1.56) | 0.97 (0.55; 1.69) | 0.89 (0.52; 1.50) |
| Born in Denmark  No vs Yes | 2.28 (1.57; 3.30) | 2.29 (1.61; 3.27) | 0.48 (0.21; 1.13) | 0.84 (0.39; 1.76) |

^1^ For each parameter, the parameter in focus was kept constant while controlling for all other variables (i.e., education, comorbidity, working status, socio-economic status, civil status, country of birth, and age)

| Table S6. Baseline characteristics for controls in the subpopulation with known screening status | | |
| --- | --- | --- |
|  | Total study population  (n = 5,104) | |
|  | Attenders  (n = 3,872) | Non-attenders  (n = 1,232) |
| Age  26-49  50-64 | 3,129 (80.8%)  743 (19.2%) | 977 (79.3%)  255 (20.7%) |
| Education  Short  Medium  High  Unknown | 488 (12.6%)  1,709 (44.1%)  1,599 (41.3%)  76 (2.0%) | *  281 (22.8%)  483 (39.1%)  383 (31.1%)  85 (6.9%) |
| Comorbidity  CCI = 0  CCI = 1  CCI = 2  CCI >2 | 3,419 (88.3%)  254 (6.6%)  161 (4.2%)  38 (1.0%) | *  1,080 (87.7%)  81 (6.6%)  42 (3.4%)  29 (2.4%) |
| Working status  Working  Senior  Other  Unknown | 3,071 (79.3%)  255 (6.6%)  501 (12.9%)  45 (1.2%) | *  749 (60.8%)  161 (13.1%)  255 (20.7%)  67 (5.4%) |
| Socio-economic status  Low  High  Unknown | 1,831 (47.3%)  1,698 (43.9%)  343 (8.9%) | *  701 (56.9.5%)  351 (28.5%)  180 (14.6%) |
| Civil status  Living alone  Living with a partner  Unknown | 931 (24.0%)  2,894(74.7%)  47 (1.2%) | *  336 (27.3%)  801 (65.0%)  95 (7.7%) |
| Born in DK  Yes  No | 3,315 (85.6%)  557 (14.4%) | *  899 (73.0%)  333 (27.0%) |

* χ²-test with 0.05 significance level.
